# Increasing adoption of minimally invasive surgery for congenital lung malformations in young infants: a single-center experience

**DOI:** 10.3389/fped.2026.1855018

**Published:** 2026-09-22

**Authors:** Rebecca Pulvirenti, Laura Valfrè, Martina Capitani, Federico Beati, Federico Scorletti, Chiara Iacusso, Barbara Daniela Iacobelli, Fabio Fusaro, Pietro Bagolan, Andrea Conforti

**Affiliations:** 1 Neonatal and Fetal Surgery Unit, Bambino Gesù Pediatric Hospital, IRCCS, Rome, Italy; 2 Catholic University of the Sacred Heart, Rome, Italy; 3 Pediatric Surgery Unit, Department of Women and Child's Health and Public Health, “Agostino Gemelli” University Hospital Foundation, IRCCS, Rome, Italy

**Keywords:** congenital lobar emphysema, congenital lung malformations, congenital pulmonary airway malformation, minimally-invasive surgery, pulmonary sequestration, thoracoscopy, thoracotomy

## Abstract

**Objectives:**

Congenital lung malformations (CLM) encompass a heterogeneous group of conditions increasingly diagnosed prenatally. While surgical resection is widely adopted, the surgical approach is progressively shifting toward minimally invasive techniques. However, concerns persist regarding the feasibility of thoracoscopy in very young infants. This study aimed to compare thoracoscopic and open surgical resection of CLM and to evaluate temporal trends in surgical approach.

**Methods:**

A retrospective review was conducted of all patients undergoing surgical resection for CLM at a tertiary pediatric referral center between January 2011 and December 2025. Patients were grouped according to surgical approach (thoracoscopy vs. thoracotomy). Temporal trends were assessed, and a subgroup analysis including patients undergoing elective anatomical lobectomy was performed to compare perioperative outcomes. Appropriate statistical tests were applied.

**Results:**

A total of 219 patients were included. Thoracoscopic resection was performed in 90/219 (41.1%) cases and thoracotomy in 122/219 (55.7%), with a small proportion undergoing alternative approaches. Temporal trend analysis demonstrated a significant increase in thoracoscopic surgery over time, rising from 25.25% in 2011–2018 to 32.65% in 2019–2021 and reaching 77.78% in 2022–2025 (*p* < 0.001). In the subgroup of patients undergoing anatomical lobectomy (*n* = 138), thoracoscopy was associated with perioperative outcomes comparable to thoracotomy, with no significant differences in operative time, length of stay, or postoperative complications.

**Conclusion:**

These findings support the progressive adoption of thoracoscopic surgery for CLM and demonstrate that, following an institutional learning curve, minimally invasive resection can be safely performed even in early infancy.

## 1 Introduction

Congenital lung malformations (CLM) represent a heterogeneous group of conditions, including congenital pulmonary airway malformation (CPAM), extra- and intralobar sequestration (EBS, IBS), congenital lobar emphysema, and bronchogenic cysts. The reported incidence of CLM has increased markedly over recent decades, rising from approximately 1 in 30,000 to 1 in 2,500 live births, largely due to advances in prenatal ultrasound (US) and improved diagnostic accuracy (1). CLMs are often detectable from the second trimester of pregnancy, allowing early assessment of lesion characteristics and their potential impact on lung development and fetal wellbeing (2). At birth, many patients are asymptomatic and do not require immediate intervention; however, these lesions carry a significant risk of recurrent pulmonary infections - affecting up to 86% of patients within the first two years of life - as well as a potential risk of malignant transformation (3–6).

Symptomatic lesions may require respiratory support, urgent surgical resection, or even extracorporeal life support in severe cases. While early surgery is generally indicated in symptomatic patients, the optimal management of asymptomatic CLMs remains controversial (7–11). Centers advocating conservative management aim to avoid unnecessary surgery and exposure to general anesthesia in asymptomatic children, instead recommending careful long-term clinical and radiological follow-up (6). Conversely, many centers favor elective surgical resection because of the risk of recurrent pulmonary infections, which may increase the technical complexity of surgery, as well as the encouraging long-term outcomes reported after early resection, including preserved pulmonary function and the absence of significant anesthesia-related sequelae in most patients (5, 12).

Despite the lack of consensus, most centers currently advocate elective surgical resection as the standard of care for asymptomatic CLM, although the optimal timing of surgery remains undefined (13–15). Surgical intervention is generally recommended before two years of age to reduce the risk of infection, but no universally accepted timing exists (16).

Surgical management has progressively evolved from open thoracotomy to thoracoscopic resection, now increasingly considered the gold standard (17). Thoracoscopy offers advantages such as reduced postoperative pain, shorter hospital stay, and improved visualization, but it remains technically challenging in small infants due to limited operative space and complex intraoperative management (18).

Since 2019, thoracoscopic resection has been increasingly adopted as the standard approach for asymptomatic CLM at our tertiary referral center. In our practice, patients typically undergo segmental resection or lobectomy at an early age (3–4 months of life) in order to minimize the risk of complications related to lesion progression.

The aim of this study is to report our institutional experience in the management of CLM, with a particular focus on the feasibility of minimally invasive surgery in young infants. Additionally, a subgroup analysis was performed to evaluate postoperative outcomes in patients undergoing anatomical lobectomy, with the goal of identifying strategies to safely implement and optimize a thoracoscopic approach.

## 2 Materials and methods

Medical records of all patients who underwent surgical resection for CLM at the Neonatal Surgical Unit of Bambino Gesù Children's Hospital between January 2011 and December 2025 were retrospectively reviewed, based on a prospectively maintained institutional database. Patients referred to our institution beyond early infancy, who had not undergone neonatal or early infant evaluation at our Neonatal Surgery Unit, were excluded from the study.

Patients were divided into two groups according to the surgical approach: thoracoscopy (Group A) and thoracotomy (Group B). To maximize comparability between the two groups, only patients who underwent elective anatomical lobectomy were included in the subgroup analyses.

To ensure consistency in surgical technique and allow a more reliable assessment of the learning curve, all thoracoscopic procedures included in the analysis were performed by a single surgeon, although this may limit the external generalizability of the findings.

Preoperative variables included gestational age at birth, birth weight, sex, prenatal diagnosis, *in utero* treatment, age at surgery, and need for emergency intervention. Intraoperative and postoperative variables included operative time, conversion to open surgery, early postoperative complications (defined as occurring within 7 days after surgery and graded according to the Clavien–Dindo classification), and length of hospital stay (19).

The study was conducted in accordance with the principles of the Declaration of Helsinki. Institutional Review Board approval was obtained, and the requirement for informed consent was waived due to the retrospective, observational nature of the study.

### 2.1 Management protocol

According to our institutional protocol, asymptomatic patients with a prenatal diagnosis of CLM undergo chest computed tomography (CT) between the second and third months of life. In cases of confirmed CLM, elective surgical resection is typically performed between the third and fourth months of life.

Following the implementation of thoracoscopy for the treatment of CLM, the choice of surgical approach in Group A was tailored to patient clinical conditions and respiratory tolerance.

Thoracoscopic resections were performed using a standardized three-trocar technique, without the use of single-lung ventilation. During the initial implementation of thoracoscopy, planned conversion to thoracotomy after vessel ligation was routinely adopted. Following completion of the learning curve, conversion was performed only when required because of intraoperative complications (e.g., bleeding) or inadequate ventilation.

Postoperatively, a chest tube is routinely left in place for 48–72 h. It is subsequently clamped and removed if no evidence of pneumothorax is detected on chest radiography. Postoperative pain is routinely assessed using the Face, Legs, Activity, Cry, Consolability (FLACC) scale, and analgesic therapy adjusted accordingly, typically consisting of intravenous paracetamol and tramadol (20).

Symptomatic patients, including those presenting with respiratory distress at birth, underwent urgent or emergent surgical intervention based on clinical status.

### 2.2 Statistical analysis

Descriptive statistics were used to summarize the study population. Continuous variables are reported as mean (standard deviation, SD) or median (interquartile range, IQR), as appropriate, while categorical variables are presented as frequencies and percentages.

Temporal trends in surgical approach were assessed using the linear-by-linear association test (chi-square test for trend). The study period was divided into three intervals according to the implementation of thoracoscopic surgery at our institution: 2011–2018, before the systematic introduction of thoracoscopy for CLM; 2019–2021, during the implementation phase; and 2022–2025, after thoracoscopy had become the standard surgical approach for suitable patients.

Data distribution was assessed using the Shapiro–Wilk test, and non-parametric tests were applied as appropriate. Comparisons between Group A and Group B were conducted using the Mann–Whitney *U* test for continuous variables and Fisher's exact test for categorical variables.

A *p*-value < 0.05 was considered statistically significant. Statistical analyses were performed using SPSS version 28.0.1.0 (IBM Corp., Armonk, NY, USA).

## 3 Results

A total of 219 patients met the inclusion criteria, including 114 males (52.05%). A prenatal diagnosis of CLM was established in 205/219 (93.61%) cases. Among these, 18/205 (8.78%) required prenatal medical treatment, while 5/205 (2.44%) underwent prenatal surgical intervention, including thoraco-amniotic shunt placement (*n* = 4) and cyst aspiration (*n* = 1). Mean gestational age at birth was 38.25 ± 1.92 weeks, and mean birth weight was 3.21 ± 0.52 kg. A total of 48/219 (21.92%) patients presented with symptoms at birth; of these, 28/48 (58.33%) had mild or transient symptoms, whereas 20/48 (41.67%) required urgent or emergency intervention. An additional three patients (1.37%) developed symptoms within the first days of life, resulting in a total of 23/219 (10.5%) emergency surgical procedures. All 23 patients undergoing emergency surgery presented with respiratory distress; among them, 3/23 (13.04%) developed pneumothorax, 6/23 (26.09%) required respiratory support, including intubation, and 4/23 (17.39%) required extracorporeal life support (ECLS). No episodes of pneumonia before surgery were documented in patients undergoing elective resection. Chest computed tomography (CT) data were available for 215/219 (98.17%) patients and were performed at a median age of 3.01 months (IQR 2.33–3.84). Based on imaging findings, 140/215 (65.12%) lesions were classified as suspected CPAM, 50/215 (23.26%) as pulmonary sequestration, 4/215 (1.86%) as congenital lobar emphysema, 2/215 (0.93%) as bronchogenic cysts, and 19/215 (8.84%) as other lesions. Details of the affected pulmonary lobes can be found in Figure 1.

**Figure 1:**
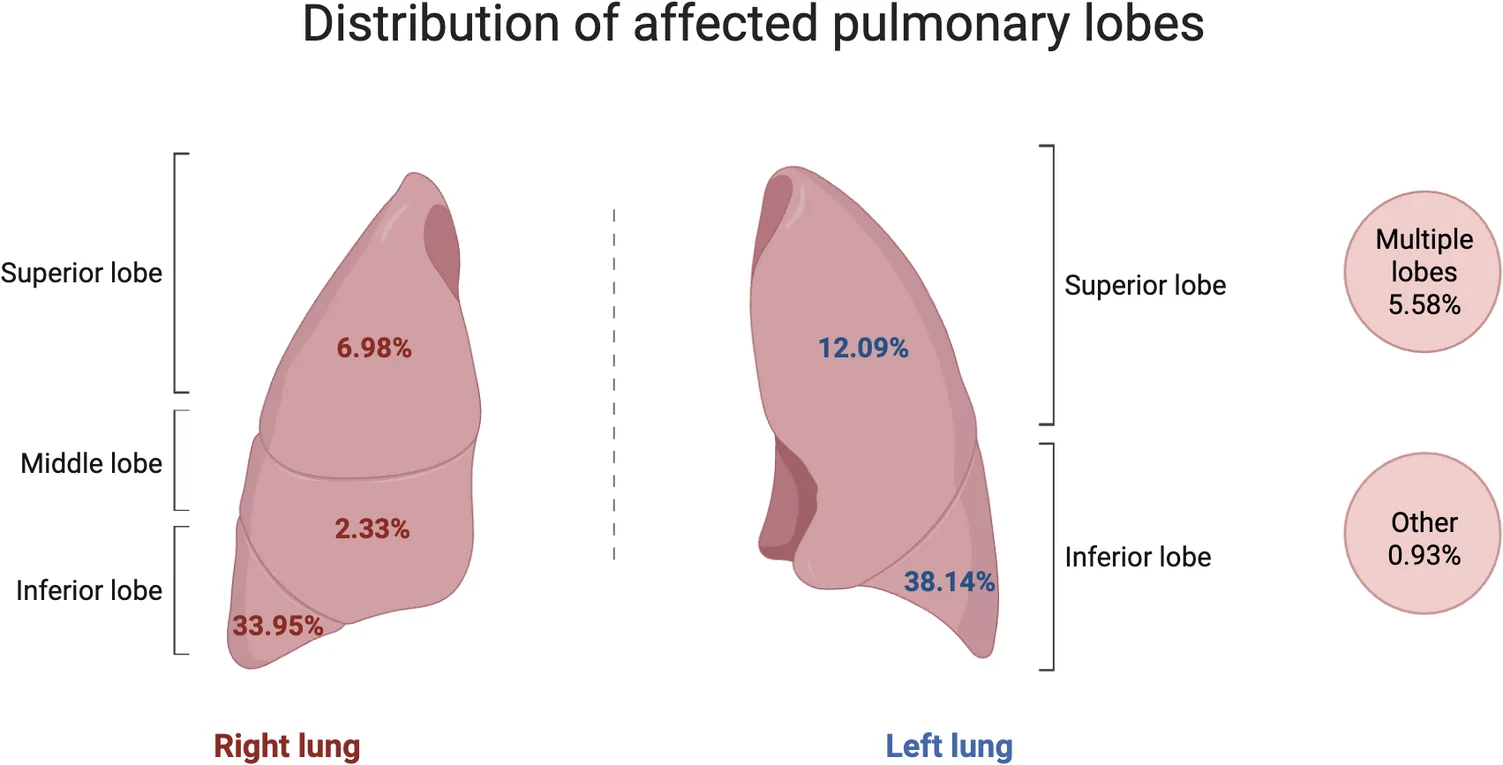
Distribution of affected pulmonary lobes.

During the study period, 90/219 (41.09%) patients underwent thoracoscopic resection and 122/219 (55.71%) thoracotomy, while 6 (2.74%) patients required a laparoscopic procedure for subdiaphragmatic pulmonary sequestration and 1 patient (0.46%) a combined thoracoscopic and open approach. Out of the 23 patients requiring an emergency surgery, 2/23 (8.69%) underwent thoracoscopic surgery, while 21/23 (91.31%) an open thoracic approach.

Conversion from thoracoscopy to thoracotomy occurred in 16/90 (17.78%) cases. The reasons for conversion included large lesion size in 4/16 (25%) cases, intolerance to pneumothorax in 1/16 (6.25%), and intraoperative challenges related to bronchial, vascular, or cystic components in 3/16 (18.75%). In the remaining 8/16 (50%) cases, complex anatomy or inflammatory changes led to prolonged operative time, prompting conversion to an open approach.

Additionally, during the early phase of thoracoscopic implementation, 7/90 (7.78%) patients underwent a planned conversion after initial thoracoscopic vessel ligation.

Histological examination confirmed CPAM in 103/219 (47.03%) patients, intralobar pulmonary sequestration in 22/219 (10.05%), and extralobar pulmonary sequestration in 33/219 (15.07%), with additional histological subtypes detailed in Figure 2. Two deaths were reported (0.91%), both in premature infants who underwent emergency surgery.

**Figure 2:**
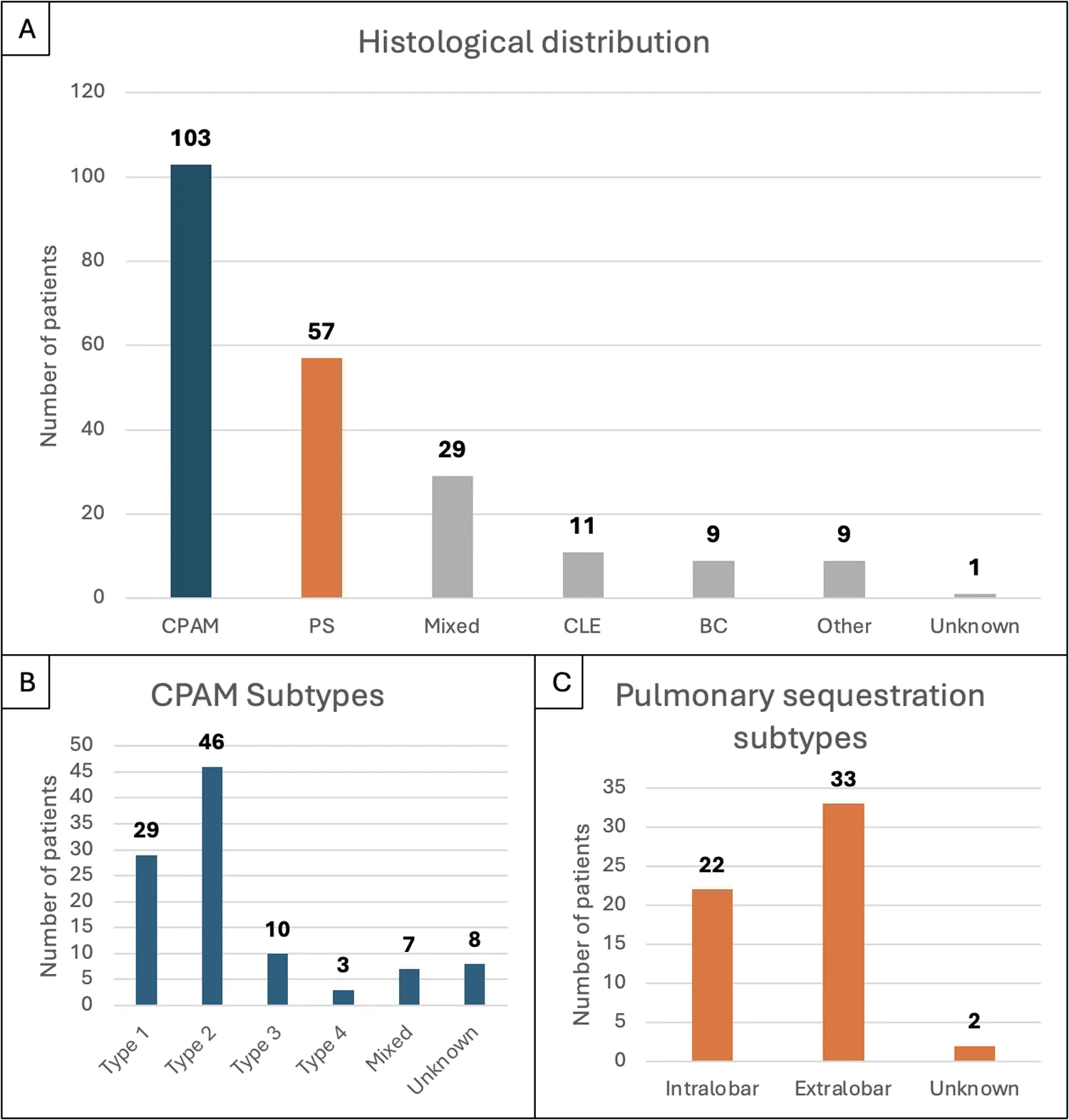
Histological distribution of congenital lung malformations. **(A)** Overall distribution of histological diagnoses in the study cohort. **(B)** Subclassification of CPAM lesions. **(C)** Subclassification of pulmonary sequestration. CLE, congenital lobar emphysema; BC, bronchogenic cyst; CPAM, congenital pulmonary airway malformation; PS, pulmonary sequestration.

Analysis of temporal trends demonstrated a progressive and statistically significant increase in the use of thoracoscopic surgery over time. Specifically, during 2011–2018 25/102 thoracoscopies were performed, compared to 75/102 open surgeries (plus 2/102 laparoscopies); between 2019 and 2021, 16/53 surgeries were thoracoscopies, while 33/53 open surgeries (plus 4/53 laparoscopies and 1/53 combined approach);finally, between 2022 and 2025, 49/64 were thoracoscopies and 14/64 open surgeries (plus 1/64 laparoscopies). The proportion of thoracoscopic procedures rose from 25.25% in 2011–2018 to 32.65% in 2019–2021, and markedly increased to 77.78% in 2022–2025 (*p* < 0.001), indicating a clear shift toward minimally invasive surgical management (Figure 3).

**Figure 3:**
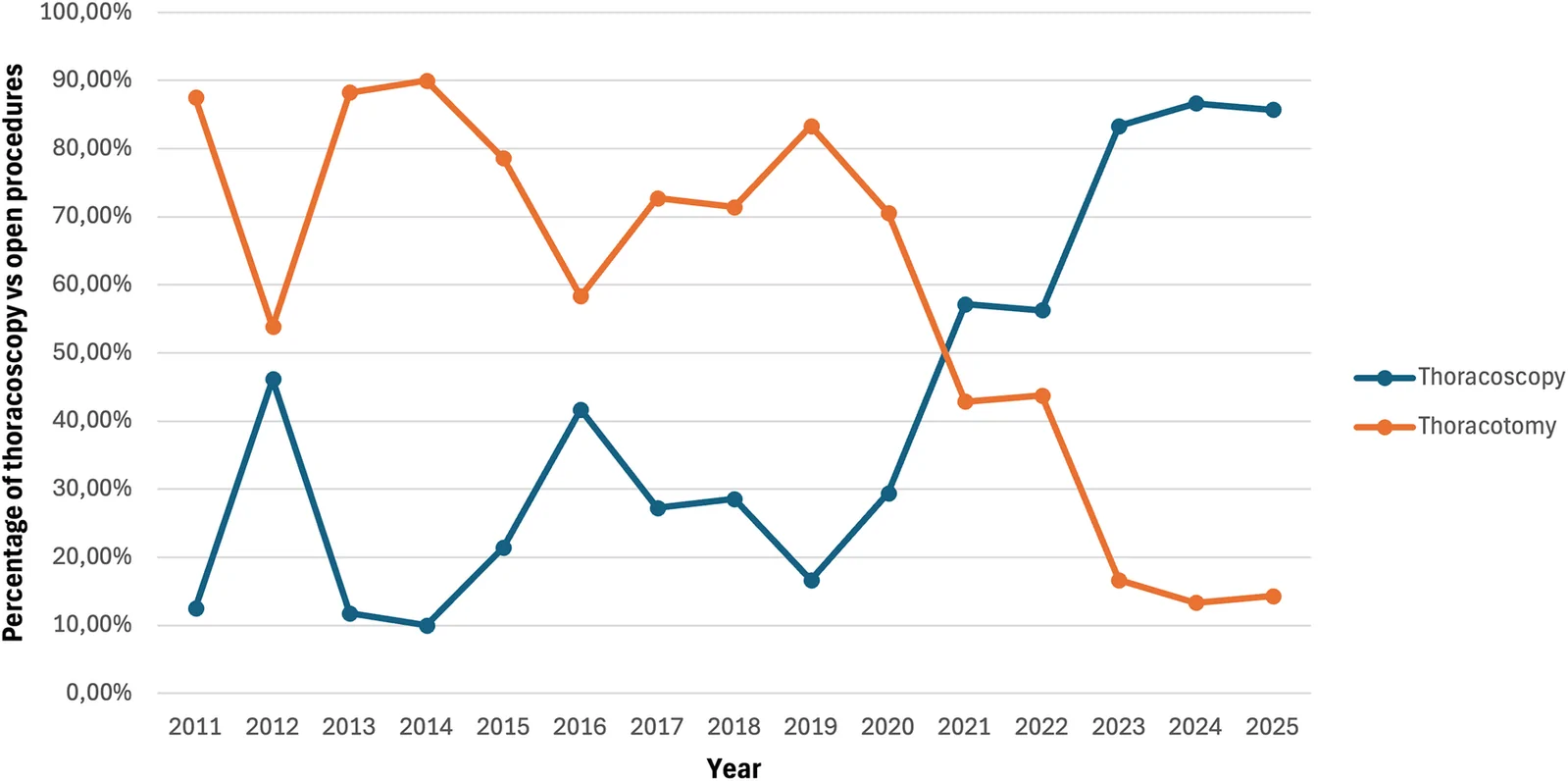
Temporal trends in surgical approach for congenital lung malformations.

Unplanned conversion to thoracotomy occurred in 8/25 (32%) patients during 2011–2018, 2/16 (12.5%) patients during 2019–2021, and 6/49 (12.24%) patients during 2022–2025. Notably, 3/8 (37.5%) unplanned conversions in the earliest period were prompted by intraoperative complications (bleeding or inadvertent cyst opening), whereas no conversions due to intraoperative complications occurred during the most recent study period. Conversely, planned conversion following thoracoscopic hilar vessel ligation was performed in 2/25 (8%) patients during 2011–2018, 4/16 (25%) patients during 2019–2021, and only 1/49 (2.04%) patients during 2022–2025.

### 3.1 Subgroup analysis

A total of 138 patients underwent elective anatomical lobectomy, including 44/138 (31.88%) in the thoracoscopic group (A) and 94/138 (68.12%) in the thoracotomy group (B) (Figure 4). No significant difference was observed in chest drain placement between the two groups (39/44 vs. 91/94, *p* = 0.11).

**Figure 4:**
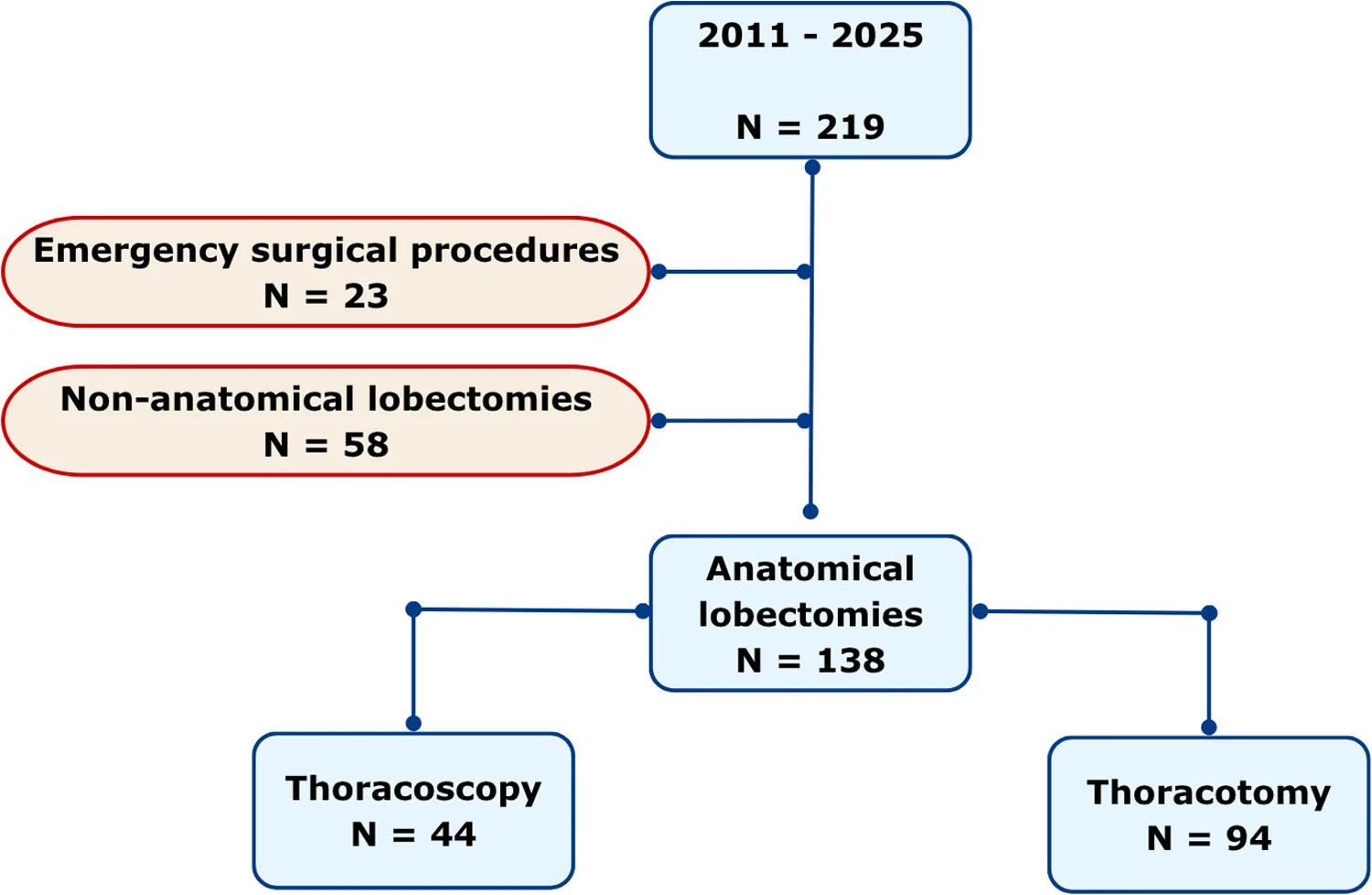
Study flow diagram showing patient selection for the subgroup analysis.

Age at surgery was significantly higher in patients undergoing thoracoscopic resection compared to thoracotomy [median 4.39 months [IQR 3.62–6.03] vs. 3.75 months [IQR 2.92–4.70], *p* = 0.007]. No statistically significant differences were found in operative time, length of hospital stay, need for NICU admission, or postoperative complications, including severe complications (Clavien–Dindo ≥ 3) (Table 1).

**Table 1:** Perioperative outcomes following thoracoscopic versus open anatomical lobectomy.

	Thoracoscopy (*N* = 44)	Thoracotomy (*N* = 94)	*p*-value
Age at surgery, months	4.39 (3.62-6.03)	3.75 (2.92–4.70)	0.007
Operative time, minutes	166.50 (139.50–215)	151 (134.50–171.50)	0.063
Chest drain placement, N(%)	39 (88.64)	91 (96.81)	0.11
Length of stay, days	5 (4–9)	6 (5–8)	0.14
NICU admission, N(%)	1 (2.27)	8 (8.51)	0.27
Any complication, N(%)	15 (34.09)	22 (23.40)	0.22
Clavien-Dindo ≥3, N(%)	9 (20.45)	13 (13.83)	0.33

Values are presented as median (interquartile range), unless otherwise specified.

## 4 Discussion

Our findings demonstrate a clear temporal shift toward thoracoscopic resection of congenital lung malformations (CLM), with minimally invasive surgery becoming the dominant approach in the most recent period. Importantly, thoracoscopy was associated with perioperative outcomes comparable to thoracotomy, despite the very young age at intervention.

The growing consensus regarding the benefits of minimally invasive surgery has been progressively supported by numerous studies demonstrating its feasibility and favorable perioperative outcomes. However, the steep learning curve associated with pediatric thoracoscopy may still limit its widespread adoption in patients with CLM (21, 22). In this context, our results clearly illustrate the evolution of indications and utilization of thoracoscopy over the study period, likely facilitated by the relatively high case volume at our center.

When compared with previous literature, our findings provide additional insight into the maturation of thoracoscopic practice. Laje et al. reported significantly longer operative times for thoracoscopic surgery compared to thoracotomy (23). In contrast, operative times in our series did not differ significantly between groups, possibly reflecting the increased adoption and standardization of thoracoscopy in pediatric surgery over time. The learning curve likely played a key role in this process, contributing to improved efficiency as experience increased. At the same time, the broader inclusion criteria for thoracoscopy in the most recent years (2022–2025) led to the inclusion of more complex cases, which are inherently associated with longer operative times. Similarly, Park et al. demonstrated a progressive expansion of indications and a reduction in conversion rates over a 10-year period (24). Our findings extend these observations to a younger population, suggesting that these advances are also applicable to patients younger than 6 months of age, despite the additional surgical and anesthetic challenges.

The issue of conversion to open surgery remains an important consideration when evaluating the feasibility of thoracoscopy in this setting. Seong et al. identified younger age and lower body weight as potential risk factors for conversion on univariate analysis, although only previous pneumonia remained significant in multivariate analysis. In our series, the conversion rate was slightly higher than those reported in previous series (25). This finding may be partially explained by the referral nature of our center, which serves a large catchment area and may therefore include a higher proportion of complex cases and patients with larger lesions. In addition, during the early phase of thoracoscopic implementation, planned conversions were occasionally performed but were subsequently abandoned in favor of a fully thoracoscopic approach.

Another manifestation of the learning curve is reflected in the age difference observed between the thoracoscopic and thoracotomy groups. During the early implementation phase, slightly older infants were preferentially selected for thoracoscopic anatomical lobectomy, whereas this selection criterion was progressively abandoned as surgical experience increased. Consequently, thoracoscopy has become the standard approach for suitable patients at our institution, irrespective of age.

The timing of surgical intervention in CLM remains a matter of ongoing debate. Our results contribute to this discussion by supporting the feasibility of early surgery, even in infants younger than 6 months of age. This is consistent with the findings of Nakajima et al., who reported a relatively lower percent vital capacity and higher ratio of residual volume to total lung capacity (RV/TLC) in children undergoing surgery at a later age, suggesting a potential impact of timing on mid- and long-term outcomes (26). Similarly, Pio et al. reported a higher forced expiratory volume in one second/forced vital capacity (FEV1/FVC) ratio in patients undergoing surgery before five months of age, although the difference did not reach statistical significance (27). Although surgery before two years of age has become widely accepted, evidence specifically addressing outcomes in younger infants is still limited. In this regard, previous studies comparing open and minimally invasive lobectomy in infants have highlighted several technical advantages of early intervention, including reduced inflammatory changes, less hilar lymphadenopathy, and smaller bronchi and vessels that are more amenable to dissection and sealing devices or clips (23, 28). While large cohort studies are needed to further clarify the optimal timing of surgery, our findings support the feasibility of minimally invasive surgery even at a very young age.

Consistent with prior literature, we observed no significant differences in postoperative complications between thoracoscopic and open approaches. These findings align with those reported by Raymond et al. in a propensity-matched cohort, further supporting the safety of thoracoscopic surgery in this population (29).

Beyond short-term outcomes, the potential long-term benefits of minimally invasive surgery should also be considered. Open thoracotomy has been associated with musculoskeletal sequelae, including scoliosis and winged scapula (30–33).

Although not assessed in the present study, previous reports in other congenital conditions, such as esophageal atresia, have demonstrated improved musculoskeletal outcomes and a reduced incidence of thoracogenic scoliosis following minimally invasive approaches (34). Similarly, we previously reported a high rate of musculoskeletal deformities in patients operated on for CLM. Despite the presence of pulmonary symptoms and musculoskeletal sequelae, overall outcomes remained favorable. These complications are often pauci-symptomatic; however, the use of less invasive approaches, such as minimally invasive axillary muscle-sparing thoracotomy or thoracoscopy, may help reduce this burden (35). Future studies should specifically address these long-term outcomes in patients with CLM.

While our findings support the feasibility and safety of thoracoscopic surgery even in very young infants, they should be interpreted in light of several limitations, including the learning curve. First, the retrospective design inherently introduces the risk of selection bias. In particular, the choice of surgical approach was not randomized but evolved over time, reflecting increasing institutional experience and broader adoption of minimally invasive techniques. This may have resulted in confounding by indication, as thoracoscopy was initially reserved for selected patients considered more suitable for a minimally invasive approach, with indications progressively broadening over time. As a result, differences between groups may be partially influenced by temporal trends and evolving patient selection rather than the surgical approach itself. Second, although patients were broadly comparable, unmeasured confounders - such as differences in lesion complexity or surgeon preference - may have influenced both treatment allocation and outcomes. Propensity score matching was not performed, limiting our ability to further account for these potential differences between groups. Third, the sample size, particularly in subgroup analyses, may limit the statistical power to detect small differences in outcomes. Furthermore, the lack of long-term follow-up precludes assessment of pulmonary function, recurrence, and musculoskeletal sequelae. Finally, all thoracoscopic procedures were performed by a single surgeon, which ensured consistency in surgical technique but limits the external generalizability of the findings. This limitation is further inherent to the single-center design and the high-volume tertiary referral setting, and our results may therefore not be directly generalizable to other settings.

Despite these limitations, this study represents one of the largest comparisons of thoracoscopic versus open surgical management of CLM in a cohort of infants. A key strength lies in the comparability of patients in terms of age, size, lesion type, and disease severity, despite the temporal evolution in surgical approach. Overall, our findings provide clinically relevant evidence supporting the progressive adoption of thoracoscopic surgery for CLM, even in very young patients.

## Data Availability

The raw data supporting the conclusions of this article will be made available by the authors, without undue reservation.
